# Importance of Long-Term Cycles for Predicting Water Level Dynamics in Natural Lakes

**DOI:** 10.1371/journal.pone.0119253

**Published:** 2015-03-10

**Authors:** Jorge García Molinos, Mafalda Viana, Michael Brennan, Ian Donohue

**Affiliations:** 1 School of Natural Sciences, Department of Zoology, Trinity College Dublin, Dublin, Ireland; 2 Trinity Centre for Biodiversity Research, Trinity College Dublin, Dublin, Ireland; 3 Center for Environmental Biology and Ecosystem Studies, National Institute for Environmental Studies, Tsukuba, Ibaraki, Japan; University of Vigo, SPAIN

## Abstract

Lakes are disproportionately important ecosystems for humanity, containing 77% of the liquid surface freshwater on Earth and comprising key contributors to global biodiversity. With an ever-growing human demand for water and increasing climate uncertainty, there is pressing need for improved understanding of the underlying patterns of natural variability of water resources and consideration of their implications for water resource management and conservation. Here we use Bayesian harmonic regression models to characterise water level dynamics and study the influence of cyclic components in confounding estimation of long-term directional trends in water levels in natural Irish lakes. We found that the lakes were characterised by a common and well-defined annual seasonality and several inter-annual and inter-decadal cycles with strong transient behaviour over time. Importantly, failing to account for the longer-term cyclic components produced a significant overall underestimation of the trend effect. Our findings demonstrate the importance of contextualising lake water resource management to the specific physical setting of lakes.

## Introduction

The global anthropogenisation of Earth’s biomes and the appropriation of its natural resources under increasing climate uncertainty are complex and novel environmental and socioeconomic challenges for society [1–4]. Water is recognised widely as the most essential of natural resources because of its importance to every facet of human life, limited supply and unequal distribution [5]. Sustainable use of global water resources is being hindered by the current intensification of the Earth’s water cycle with atmospheric warming [6]. Providing for an ever-growing demand of water in an efficient and secure manner requires, therefore, intensive technological intervention with its own profound environmental implications [7, 8]. An estimated 80% of the global human population inhabit areas with threatened water security [8]. Consequently, sustainable water use requires increasingly adaptive and integrated management strategies capable of acting rapidly by incorporating current resource uncertainty [9], while allocating existing resources under competing uses by integrating environmental, human and technological factors [10].

Lakes contain 77% of the liquid surface fresh water on Earth [11], provide multiple essential ecosystem services [12] and support extremely high [13], yet fragile [14], levels of biodiversity. Water level dynamics comprise one of the most important physical processes in lakes with significant socio-economic and environmental implications. Extreme high water episodes or significant upward trends in water levels can culminate in shoreline damage [15], while prolonged decreasing water levels may generate water quality issues [16] and impact the delivery of lake ecosystem services [17]. Water level fluctuations regulate the dynamics of biological communities [18], the water and nutrient balances of lakes, the interaction between littoral and pelagic zones and the flux of organic material [19]. Extremely high or low water levels can alter whole ecosystems dramatically, by, for example, altering patterns of sediment deposition and inducing shifts in their trophic state [20]. Further, the functioning of lake ecosystems is driven by fluctuations in water levels that occur at a variety of temporal scales, driven by weather patterns, climatic processes and human disturbance [21, 22]. For example, surrounding ecosystems forming the aquatic-terrestrial interface rely strongly on the seasonal and periodic fluctuations in water levels [23]. Maintenance and restoration of natural water level regimes is, therefore, crucial to enhance water quality and biodiversity and to preserve the multiple ecosystem goods and services provided by lakes [24].

Active management of water levels is advocated as a socio-economic and environmentally-balanced solution to lake water resource management [25]. However, uncertainty and temporal variability in water resource availability across a range of temporal scales [26] makes rational planning and management based on water level regimes highly complex. Natural water level fluctuations in lakes encompass both seasonal and cyclical components superimposed on long-term trends and stochastic noise. These are subject to frequent temporal shifts and changes linked to the nonlinear, stochastic or transient effects of external factors such as global climate forcing [27], anthropogenic activities [28] and their interaction [22]. Such variation in the temporal patterning of environmental variation can be a key determinant of biotic community dynamics and susceptibility to disturbance [29, 30]. Whereas seasonal and cyclic components are obviously important to resource management, a coherent management strategy needs to be based ultimately on long-term resource availability. This requires application of flexible analytical tools that allow explicit incorporation of seasonal and possibly other more long-term cyclic components across a range of temporal scales. Moreover, the analysis of long-term datasets also generally involves making important decisions about how to deal with missing data. Commonly, this involves choosing between alternative imputation procedures which can have significant influence on the models that are ultimately produced. Bayesian inference offers a flexible framework that can help to avoid these important problems, including the possibility of using incomplete series without having to recur to imputation procedures and the possibility of fitting models without prior knowledge of the periodicity associated with harmonics. For these reasons, we used Bayesian harmonic regression models to (i) explore the importance of cyclic components in confounding estimation of long-term (22–37 years) directional trends in water levels in 28 natural lakes in Ireland and (ii) analyse the magnitude of trends in changing water levels.

## Materials and Methods

### Water level series

We quantified long-term monthly water level series (ranging from 1974–2012) for 28 natural lakes in Ireland (Table 1) from relative mean daily water levels recorded at gauge stations on each lake. We set one complete week of daily data as the minimum required for the computation of the mean water level in a month. Otherwise, a month was considered as a missing observation in the final series. We based this criterion on the highly significant (*α* < 0.001) water level autocorrelations found in all series as determined by the Durbin-Watson test [31] under the null hypothesis of no temporal autocorrelation in the series at a 30-day lag. The resulting proportion of missing observations accounted for 2.7 ± 2.9% of the time series (comprising [mean ± SD] 31 ± 5 continuous years for each lake).

**Table 1:** List of studied lakes and associated water level series characteristics, with information on their surface area, mean depth (where known) and current (2008–2009) annual abstraction volumes.

Lake	Latitude / Longitude (decimal degrees)	County	Start / End	% Missing values	Abstracted volume (hm^3^ yr^-1^)	Surface area (ha)	Mean Depth (m)
Akibbon (Ak)	55.013N-7.892W	Donegal	11-1975 / 5-1997	0	0	44.5	
Anure (An)	55.007N-8.276W	Donegal	11-1975 / 1-2012	0.2	1.67	132.6	4.5
Bawn (Ba)	54.047N-6.91W	Monaghan	10-1976 / 2-2012	1.9	0.95	30.4	
Cullin (Cu)	53.966N-9.141W	Mayo	9-1983 / 1-2009	4.3	0	1019.3	5
Cutra (Ct)	53.028 N-8.772 W	Galway	9-1976 / 3-2012	4.9	0	382	4
Derryclare (De)	53.464N-9.803W	Galway	4-1979 / 3-2012	1.3	0	222.5	6.9
Derrygooney (Dr)	54.042 N-6.942 W	Monaghan	10-1976 / 12-2009	2.8	0	23.2	
Dromore (Dr)	54.082N-7.087W	Monaghan	11-1975 / 2-2012	6.9	0	60.5	2.9
Egish (Eg)	54.058N-6.774W	Monaghan	3-1975 / 3-2009	2.4	0	111.4	3.3
Emy (Em)	54.347N-6.937W	Monaghan	10-1981 / 1-2012	3	0.3	52.4	
Eske (Es)	54.687N-8.052W	Donegal	9-1977 / 3-2012	0	1.09	385.2	7.3
Fad (Fa)	55.234N-7.376W	Donegal	9-1978 / 3-2012	11.4	1.4*	40.2	5.8
Feeagh (Fe)	53.925N-9.572W	Mayo	3-1976 / 02-2012	2.5	0	393.1	14.5
Fern (Fr)	55.054N-7.727W	Donegal	9-1976 / 7-1999	0.4	0	180.3	1
Gartan (Ga)	55.003N-7.906W	Donegal	11-1975 / 7-1999	0	0.1	202.8	4
Garty (Gr)	53.925N-7.583W	Cavan	10-1977 / 9-2000	1.8	1.02	82.2	6.7
Gill (Gi)	54.249N-8.439W	Sligo	3-1975 / 2-2012	0.5	4.9	1375.3	5
Gleincmurrin (Gl)	53.308N-9.498W	Galway	7-1976 / 11-2011	2.1	0	162.3	3.8
Gowna (Go)	53.866N-7.544W	Cavan	6-1976 / 2-2012	3.7	0.09	1146.7	3.7
Inchiquin (In)	52.951N-9.083W	Clare	11-1976 / 12-2011	2.6	0.17	107.3	10.1
Islandeady (Is)	53.838N-9.372W	Mayo	10-1976 / 5-1996	0.8	0	138.5	3.2
Lickeen (Li)	52.963N-9.245W	Clare	1-1976 / 8-2003	1.5	1.68	83.9	3.9
Muckno (Mu)	54.1N-6.682W	Monaghan	2-1976 / 1-2012	4.2	0.13	354.3	5.4
Nadregeel (Na)	53.883N-7.162W	Cavan	11-1976 / 12-2005	8	1.02	84.3	2.4
Oughter (Ou)	54.038N-7.433W	Cavan	10-1977 / 2-2012	0.2	0	658.2	2.6
Sillan (Si)	54.007N-6.947W	Cavan	11-1974 / 1-2006	0.5	0	161.5	4.8
Skeagh (Sk)	53.951N-7.007W	Cavan	6-1975 / 2-2012	0.9	1.21	61	2.2
White (Wh)	54.114N-6.972W	Monaghan	4-1976 / 12-2009	1.5	0.26	53.6	1

* 64% from unspecified mixed sources (surface/ground water).

### Bayesian harmonic regression

We used Bayesian harmonic regression (HREG) models with a linear component to identify the long-term trends in water levels in each lake. Harmonic regression was used to capture the seasonal and periodic cycles in water level series and provide robust estimates of trends. Missing values were incorporated into the models as unknowns and estimated from the posterior distribution following Bayes’ Theorem [32]. This is a clear advantage over frequentist approaches where missing observations need to be imputed *a priori*.

We set the sample distribution of water levels as drawn from a gamma distribution related to the linear predictor *Y*_*t*_ using a log-link function

$$Y_{t}=\beta_{0}+\beta_{1}t+\sum_{k=1}^{K}\left(\alpha_{k}\cos\frac{2\pi t}{P_{k}}+\rho_{k}\sin\frac{2\pi t}{P_{k}}\right)+\beta_{AR}Y_{t-1}+e_{t}$$

where *β*_*0*_ is the intercept, *β*_*1*_ the temporal trend, and *β*_*AR*_ the autoregressive (AR) coefficient. The AR component was introduced to account for strong monthly temporal autocorrelation in the series. The *K* harmonics in the model were expressed as a combination of sine and cosine waves with amplitude defined by the coefficients *α*_*k*_ and *ρ*_*k*_, and period *P*_*k*_ denoting the time required to complete one cycle of a harmonic. Normal distributions with mean zero and variance 10^-6^ were assigned to the regression coefficients and intercept, while the autoregressive coefficient was defined by a uniform distribution between-1 and 1 (boundary conditions are required for a stationary process).

Because the seasonal pattern of water level fluctuations at the annual periodicity is strong and well-known in temperate lakes, taking place in winter (high water) and summer (low water) in our study lakes, our first model alternative comprised a single harmonic (*K* = 1) chosen to contain just the annual seasonality with a prior drawn from a uniform distribution between 6 and 18 months: *P*_*1*_ ~ *U* (6, 18). A second alternative (*K* = 2) was given by adding a second harmonic to the seasonal model, accounting for non-seasonal long-term cycles, was described by *P*_*2*_ ~ *U* (24, *N*), where *N* indicates the total length of the series. Inter-annual as well as multi-decadal water level cycles are common in natural lakes, usually associated with natural climatic inter-decadal oscillations [27, 33]. We therefore considered a third model alternative with three harmonics (*K* = 3) including an annual, an inter-annual *P*_*2*_ ~ *U* (24, 132) and an open-prior inter-decadal harmonic *P*_*3*_ ~ *U* (144, *N*). A fourth set of model alternatives comprised a null model that consisted of only the trend component (*K* = 0). Model selection was made on the basis of convergence and the lowest deviance information criterion (DIC [34]). Given our *a priori* interest in exploring the nature and relative importance of long-term cycles in lake water level dynamics, we selected models with the higher number of harmonics where there were two or more competing models (i.e., where ΔDIC < 4) for any given lake. We also examined whether the incorporation of inter-annual and / or inter-decadal harmonics in these models increased model performance for estimation of the trend coefficient, compared with the competing models from those lakes that accounted for seasonality alone. Model performance was tested both in terms of precision (i.e., the trend coefficient itself) and accuracy (i.e., absolute magnitude of the 95% credible interval associated with the coefficient).

Convergence of the HREG models was verified using the Heidelberg statistic and visual inspection of the trace plots after running two chains for 10^5^-10^6^ iterations with a thinning of 10 and 10^4^ burn-in values. Visual assessment of model residuals was conducted after model convergence to ensure compliance of the selected model with statistical assumptions. HREG models were run using R version 2.14.1 [35] and JAGS [36] software.

## Results and Discussion

**Fig 1:**
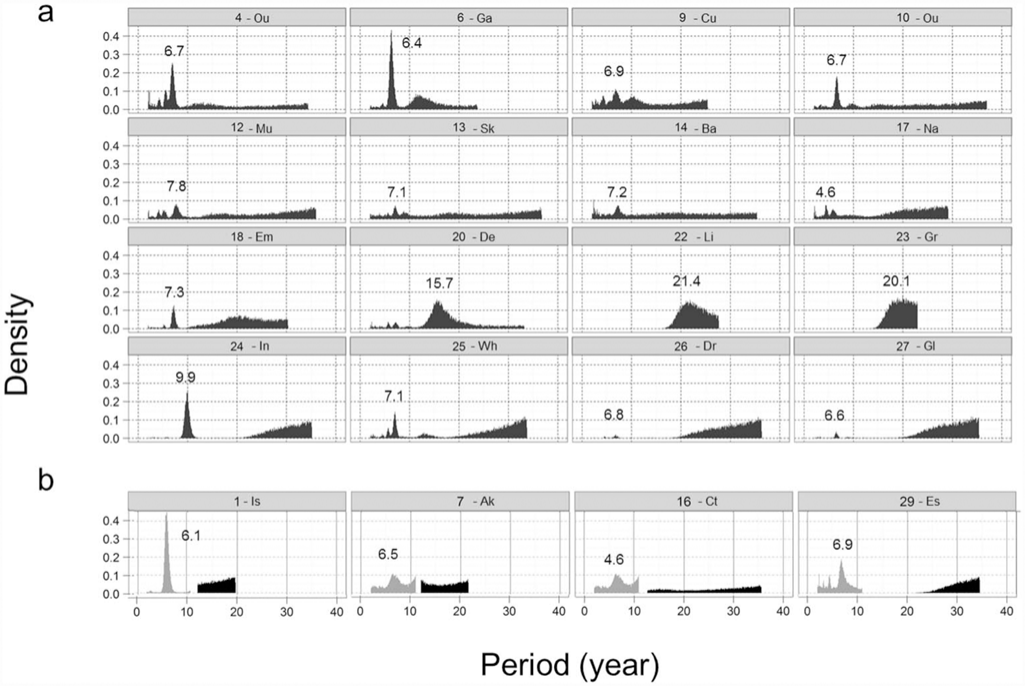
Posterior distributions of long-term cycle periods for lakes best described by multi-harmonic models. Posterior distributions of long-term (>2 year) cycle periods on monthly water levels for lakes best described by models containing (a) two harmonics with a seasonal [*P*_*1*_ ~ *U* (6, 18)] and a long-term harmonic [*P*_*2*_ ~ *U* (24, *N*)] and (b) three harmonics comprising a seasonal [*P*_*1*_ ~ *U* (6, 18)], an inter-annual harmonic [in grey; *P*_*2*_ ~ *U* (24, 132)] and an inter-decadal [in black; *P*_*3*_ ~ *U* (144, *N*)] harmonic. Numbers inside the panels indicate the periodicity corresponding to the main peak in each posterior distribution. Truncated ends of the density functions correspond to either the limits of the prior interval or the end of the time series. Lake abbreviations as in Table 1.

Our HREG models located consistently a very strong annual seasonal component in all lakes associated with extremely tight credible intervals (Table 2; S1 Table in Supporting Information). This was expected, as the seasonality of these lakes tends to be well-defined with summer minima and winter maxima. However, we found considerable variation in the structure of the best HREG model among the lakes. Though the incorporation of the annual seasonal harmonic consistently improved strongly each of the models, the best model for some lakes (43%) comprised only a simple seasonal harmonic while others performed better with models incorporating two (50%) or even three (7%) harmonics (Table 2 & S1 Table). Long-term periodicities for those lakes best described by models with two- or three-harmonics frequently displayed multimodal posterior distributions (Fig. 1), resulting in mean estimates subject to strong uncertainty as indicated by their much wider credible intervals (Table 2). This is suggestive of dynamic cyclic behaviour. Nonetheless, posterior distributions peaked locally with enough regularity to suggest the likely existence of cycles associated with specific periodicities, particularly in the 4–10 year range (Fig. 1). On the inter-decadal scale, the posterior distributions of some models suggested the likely presence of oscillations with periodicities approximating 15–25 years, though these were defined more broadly than the inter-annual peaks. A clear increase in density building up progressively from a periodicity of approximately 25 years, truncated by the limit imposed by the series length, was also identifiable in some lakes suggesting another diffuse but strong signal activity at very low frequencies (Fig. 1).

**Table 2:** Summary of the best HREG models, with their respective DIC values and relative DIC differences compared to the null model (i.e., no harmonics; ΔDIC) for each of the study lakes, and description of the trend mean coefficient *β*_*1*_, seasonal P_1_: *U* (6 months, 18 months) and cyclic P_2_: *U* (24, 132) and P_3_: *U* (144, N) components along with their corresponding 95% credible intervals (in parentheses).

Lake	DIC (model)	ΔDIC	*β*_*1*_ (x10^-4^)	P_1_	P_2_	P_3_
Akibbon (Ak)	-34.2	21.8	0.1 (-1.9, 2.2)	12.04 (11.92, 12.15)	84.6 (28.61, 130.42)	202.98 (145.99, 256.8)
Anure (An)	-167.4	19.3	-0.1 (-0.7, 0.6)	11.99 (11.95, 12.04)		
Bawn (Ba)	-71.6	64.6	-1.1 (-2, -0.3)	11.97 (11.94, 12)	223.53 (30.74, 416.57)	
Cullin (Cu)	193.8	71.5	-0.3 (-2.1, 1.4)	12.01 (11.96, 12.05)	154.32 (32.59, 299.34)	
Cutra (Ct)	-55.1	57.6	-0.2 (-1.2, 0.7)	12 (11.96, 12.03)	75.6 (27.77, 128.67)	306.89 (153.79, 423.09)
Derryclare (De)	-237.7	45.3	-1.4 (-2.4, 0.4)	12 (11.95, 12.04)		
Derrygooney (Dr)	-267.4	114.5	-1.9 (-3.2, -0.6)	11.98 (11.95, 12)	208.62 (49.52, 379.35)	
Dromore (Dr)	-156	113.5	-0.3 (-2.1, 1.2)	11.97 (11.94, 11.99)	350.76 (109.99, 433.07)	
Egish (Eg)	-383.3	84.8	-1.2 (-2, -0.4)	11.97 (11.94, 12.01)		
Emy (Em)	9.8	72.1	3.1 (0.9, 5)	11.97 (11.93, 12.01)	235.84 (65.28, 358.14)	
Eske (Es)	-537.3	37.3	-0.1 (-1.1, 1)	12.01 (11.97, 12.05)	77.54 (27.78, 126.37)	369.61 (297.79, 413.25)
Fad (Fa)	-517.9	7.7	-0.8 (-1.6, 0.1)	12 (11.95, 12.05)		
Feeagh (Fe)	-510.2	67.1	-1.6 (-2.4, -0.8)	11.98 (11.95, 12.01)		
Fern (Fr)	22.2	73.6	-0.3 (-2.6, 2.1)	12.03 (11.98, 12.08)		
Gartan (Ga)	-188.6	41.5	-1.7 (-3.7, 0.2)	12.03 (11.96, 12.1)	130.63 (45.83, 272.17)	
Garty (Gr)	-193.2	25.3	4 (1.4, 6.9)	12.02 (11.95, 12.09)	234.41 (187.45, 273.84)	
Gill (Gi)	-14	13.3	-0.3 (-1, 0.3)	11.98 (11.94, 12.02)		
Gleincmurrin (Gl)	-125.7	62	-0.1 (-1.4, 1.2)	11.99 (11.95, 12.03)	343.52 (74.89, 422.27)	
Gowna (Go)	39.8	172.6	-1.8 (-2.9, -0.7)	11.97 (11.95, 11.99)		
Inchiquin (In)	-94.4	111.5	-0.5 (-1.8, 0.7)	11.99 (11.96, 12.02)	294.51 (109.14, 418.84)	
Islandeady (Is)	-1.9	47.8	-4.6 (-8.3, -0.7)	12.03 (11.96, 12.09)	74.05 (44.61, 112.28)	194.57 (146.95, 234.25)
Lickeen (Li)	-141.4	24.6	-3.4 (-5.3, -1.7)	12.01 (11.95, 12.07)	272.42 (219.52, 327.87)	
Muckno (Mu)	95.8	52.6	-2.6 (-3.8, -1.1)	11.99 (11.94, 12.04)	253.45 (43.44, 426.77)	
Nadregeel (Na)	-160.5	78.8	-1.1 (-2.5, 0.5)	12.01 (11.97, 12.05)	218.52 (33.95, 345.55)	
Oughter (Ou)	527.3	168.2	-1.5 (-3.1, 0.1)	11.98 (11.96, 12)		
Sillan (Si)	-192.3	125	-0.3 (-1.4, 0.8)	12 (11.97, 12.03)		
Skeagh (Sk)	-234.4	15	-0.3 (-1, 0.4)	11.99 (11.95, 12.03)	254.66 (39.11, 435.28)	
White (Wh)	54.7	119.1	-1 (-3.2, 0.9)	11.97 (11.94, 12)	278.2 (53.83, 402.14)	

See S1 Table for details on competing models.

Evidence exists of inter-annual cycles associated with water level regimes in natural lakes linked to changes in regional climate driven primarily by variability in atmospheric teleconnections (e.g. [37]). In the North Atlantic region, the North Atlantic Oscillation (NAO) represents the foremost mode of climate variability exerting a strong influence on winter temperatures and precipitation over most of Europe[38]. Though the NAO exhibits important inter-annual and inter-decadal variability alternated with periods in which circulation patterns persist for several years [39], it has its main spectral peaks at periodicities within the 2–4 and 6–10 year bands [40]. These are in good agreement with the inter-annual periodicities observed in the posterior distributions of our models. Inferring causality from multi-decadal variability is more difficult because the need for series that are long enough to resolve the timescales of interest. There is, however, some evidence for the modulation of regional climate systems by global phenomena at time scales comparable to those observed in some of our HREG models [41]. For example, solar cycles, involving periodic changes in solar radiation, have been related to observed multi-decadal periodicities in environmental processes such as river flows, lake water levels and droughts [42].

**Fig 2:**
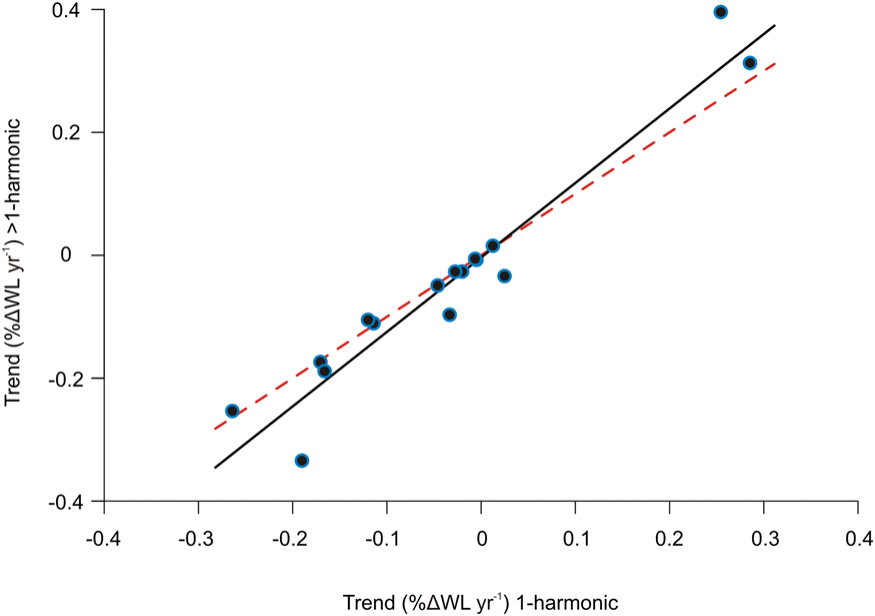
Comparison between trend estimates using a single or a multi-harmonic model. Long-term trends in mean lake water levels estimated using a single seasonal harmonic model (x-axis) or a model incorporating seasonality and inter-annual / multi-decadal cycles (y-axis) for lakes better described by a multi-harmonic model. The solid line is the linear regression relationship; the red dotted line shows the 1:1 line.

For those lakes having two or more competing models (i.e., where ΔDIC < 4; *n* = 14), estimates from models containing only the seasonal cycle produced trend estimates that were significantly both less precise (Wilcoxon signed rank test, *p* = 0.031) and less accurate (*p* = 0.00012) than those from models incorporating inter-annual and inter-decadal cycles. Further, the absolute magnitude of trends was consistently lower in models that comprised seasonal cycles alone compared with those that incorporated inter-annual and / or inter-decadal cycles (Fig. 2; slope of the latter = 1.21, test of difference from 1:1 slope: *t*_14_ = 2.4, *p* = 0.0155). These results therefore indicate clearly an importance of including long-term cycles when quantifying and predicting trends in lake water levels and giving careful consideration to the application of common procedures for trend extraction [43].

**Fig 3:**
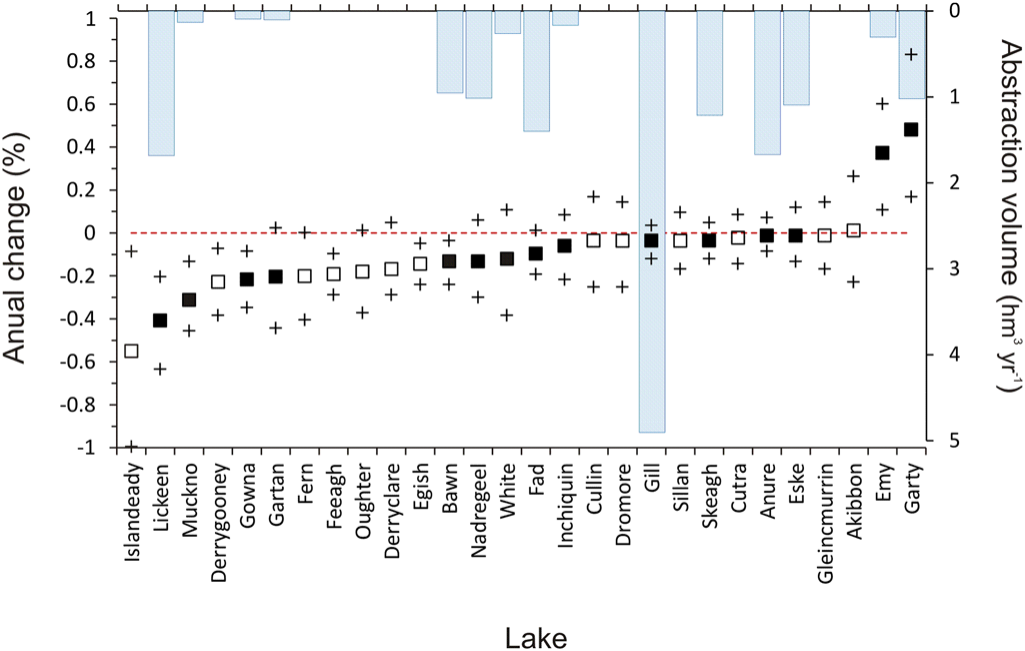
Predicted mean water level trends (squares) and 95% credible intervals (crosses) for the studied lakes. Closed symbols correspond to lakes subject to abstraction activities with background bars corresponding to their respective current (2008–2009) annual abstraction volumes. Highly probable trends are those for which their credible intervals do not intersect the red zero-threshold line.

The annual percentage change in lake mean water level over the study period, as indicated by the model trend component after accounting for seasonal and cyclic components, ranged from-0.52 to 0.48% yr^-1^ (mean ± s.e.: -0.09 ± 0.19% yr^-1^; *n* = 28). Overall, most lakes (89%) experienced negative trends, with 32% of these statistically significant (Fig. 3). Further, two of the three positive trends detected were also significant. This overall downward trend was not expected given that many of these lakes are located in areas that have experienced significant increases in precipitation in recent decades [44]. Nonetheless, we found no evidence of any spatial trend pattern among the lakes (Global Moran’s *I* = -0.093, *p* = 0.49), suggesting that anthropogenic factors are likely to be primarily responsible for the observed trends in these lakes. Water abstractions are one of the most important anthropogenic factors modifying catchment water flow and storage [45], with impacts on hydrology likely to exceed projected impacts of climate change [46]. Several of our study lakes are currently subject to water abstraction for consumption (Table 1, Fig. 3), reportedly the second most important human pressure on aquatic ecosystems in Ireland after nutrient enrichment [47]. However, we found no significant relationship between abstraction (i.e. annual volume abstracted from the lakes; Table 1) and observed water level trends (Pearson’s *ρ* = 0.13, *t*_26_ = 0.63; *p* = 0.51). The absence of such a relationship is likely a consequence of the mismatch between the temporal and spatial resolution of the data; the abstraction data comprised current (2008–2009; no historical data on abstraction was available) annual total water volumes collated for lakes but did not include abstractions from tributaries or wells, which would have had significant influence on lake water level dynamics. Further, our water level series were at monthly, rather than annual, resolutions. Collection and incorporation of such data at appropriate spatiotemporal resolutions into lake water balance models should provide improved quantification of the influence of water abstraction on the water level regimes of lakes [48].

Over decadal timescales, the observed trends in water levels could result in considerable shifts in the area, volume and shoreline length of lakes, with important ecological consequences both for the lakes themselves as well as their surrounding aquatic-terrestrial interface. Water level recession may, for example, lead to changes in substrate composition by compacting and/or redistributing littoral sediments to deeper parts of the lake [49], while water quality issues may also arise from altered sedimentation and erosive littoral processes [50, 51]. Further, with drawdown, air-exposed littoral sediment, frequently organically-enriched, can undergo complex biogeochemical reactions leading to mobilization of metal-bound phosphorus due to desiccation and oxidation of sediments and increased nitrogen loss through runoff or leaching during episodic inundations [52]. Water level fluctuations may also lead to changes in patterns of boundary mixing (i.e., the process of enhanced mixing near the lateral boundaries of a lake which affects sediment resuspension and vertical nutrient fluxes), induced mainly in stratified lakes by internal wave activity at the depth of the thermocline. Progressively declining water levels would be expected to lower the thermocline and therefore displace boundary mixing [53]. More extreme water level fluctuations can also affect stratification in freshwater lakes by facilitating vertical mixing following large drawdowns (e.g., wind forcing or nocturnal convection; [54]). All these factors can complicate effective lake management and exacerbate water quality problems by contributing to long-term eutrophication [55] and enhance the risk of lakes failing to meet specified management or policy objectives. At the other extreme, a progressive increase in mean water levels, as found for some of our lakes, will also have important management implications. For example, the flooding of terrestrial areas may reduce water quality by introducing organic matter, nutrients and chemical pollutants to lakes [56]. Increasing levels can also result in a net loss of important littoral habitat, such as reed beds, in favour of open water areas, with important implications for biodiversity, given that littoral zones provide habitat for the significant majority of biological diversity in lakes [57].

## Conclusions

Our study helps to improve our understanding of the underlying patterns of variability in water level dynamics and their associated effects in natural lakes, with clear application to adaptive water resource management under an increasingly variable climate. We show that incorporation of long-term cycles can be important for predicting trends in lake water levels, both in terms of the magnitude of the trends and the accuracy of predictions. Rapid demographic growth and uncertain hydrologic changes driven by global climate change are predicted to increase the number of people living under water shortage conditions in urban areas to 850 million by 2050 [58]; a likely highly conservative estimate as it does not account for water distribution or quality issues. The growing imbalance between water availability and demand is expected to create unprecedented ecological problems [8]. As a result, adaptive integrative strategies are needed to play an increasingly important role in directing water resource management and policy-making as governments allocate significant investment to secure water availability and ecosystem conservation.

## Supporting Information

S1 Table Summary model output for best and competing models. Summary of the mean coefficients and 95% credible intervals (in parentheses) corresponding to the best and other competing models (i.e., models within 4 DIC of the selected model; shaded) obtained for the water level series for each lake. Coefficients as described in the main text.
(PDF)
