# Supplementary material for: Importance of Long-Term Cycles for Predicting Water Level Dynamics in Natural Lakes
Source: PLoS One. 2015 Mar 10;10(3):e0119253. doi: 10.1371/journal.pone.0119253 (PMC4355075; doi:10.1371/journal.pone.0119253)
Supplement: S1 Table — Summary of the mean coefficients and 95% credible intervals (in parentheses) corresponding to the best and other competing models (i.e., models within 4 DIC of the selected model; shaded) obtained for the water level series for each lake. Coefficients as described in the main text. (PDF) [file pone.0119253.s001.pdf]

Table S1. Summary of the mean coefficients and 95% credible intervals (in parentheses) corresponding to the best and other competing models (i.e., models within 4 DIC of the selected model; shaded) obtained for the water level series for each lake. Coefficients as described in the main text.

Lake	DIC (model)	β_0	β_{WL} (x10^{-4})	β_{AR} (x10^{-2})	α_i (x10^{-3})	ρ_i (x10^{-3})	P_i	σ
Akibbon	-34.2	3.94<br>(3.64, 4.32)	0.1<br>(-1.9, 2.2)	-7<br>(-21.7, 2.3)	-24.2 (-51.6, 8.7)<br>4 (-17.4, 24.9)<br>0.6 (-18.8, 20.7)	-41.6 (-67.4, -7.4)<br>1.2 (-24.7, 24.1)<br>1.1 (-21.4, 24.2)	12.04 (11.92, 12.15)<br>84.6 (28.61, 130.42)<br>202.98 (145.99, 256.8)	25.36<br>(0.25, 91.9)
	-30.5	3.95<br>(3.63, 4.32)	0.16<br>(-1.45, 1.77)	-7.5<br>(-20.1, 4.6)	-24.3 (-51.6, 8.8)<br>-<br>-	-41.1 (-67, -7.2)<br>-<br>-	12.04(11.93, 12.15)<br>-<br>-	24.9<br>(0.26, 91.53)
Anure	-167.4	4.13<br>(3.92, 4.37)	-0.1<br>(-0.7, 0.6)	-3.4<br>(-11, 1.7)	-28.9 (-45.3, -10)<br>-<br>-	-35.5 (-52, -15.5)<br>-<br>-	11.99 (11.95, 12.04)<br>-<br>-	29.63<br>(0.75, 93.05)
Bawn	-71.6	4.03<br>(3.83, 4.22)	-1.1<br>(-2, -0.3)	-9.1<br>(-14.5, -3.6)	40.1 (13.6, 64.4)<br>-0.01 (-15.2, 15.3)<br>-	-65.9 (-83.1, -47.4)<br>0.8 (-15.4, 17.1)<br>-	11.97 (11.94, 12)<br>223.53 (30.74, 416.57)<br>-	20.12<br>(0.17, 88.56)
	-69.3	4.05<br>(3.84, 4.23)	-1.1<br>(-1.86, -0.35)	-8.2<br>(-14.1, -1.2)	39.1 (12.6, 63.5)<br>-<br>-	-65.5 (-82.6, -47)<br>-<br>-	11.97 (11.94, 12)<br>-<br>-	19.9<br>(0.14, 88.26)
Cullin	197.7	3.39<br>(3.15, 3.6)	-0.16<br>(-1.71, 1.38)	-9.5<br>(-10.3, -2.5)	82 (42.3, 118.5)<br>-<br>-	-78.5 (-107, -45.7)<br>-<br>-	12 (11.96, 12.05)<br>-<br>-	23.48<br>(0.33, 90.51)
	193.8	3.39<br>(3.16, 3.6)	-0.3<br>(-2.1, 1.4)	-8<br>(-12.8, -2.9)	82.4 (43, 117.7)<br>4.5 (-19, 27.4)<br>-	-79.1 (-107.9, -46)<br>-2.1 (-28.3, 23.1)<br>-	12.01 (11.96, 12.05)<br>154.32 (32.59, 299.34)<br>-	22.92<br>(0.34, 90.01)
Cutra	-59.4	3.88<br>(3.67, 4.07)	-0.1<br>(-0.8, 0.7)	-5.7<br>(-11.4, -0.1)	54.1 (28.7, 77.1)<br>-<br>-	-52.4 (-71.4, -30.9)<br>-<br>-	11.99 (11.96, 12.03)<br>-<br>-	26.18<br>(0.66, 91.95)
	-55.1	3.86<br>(3.68, 4.05)	-0.18<br>(-1.19, 0.71)	-5.9<br>(-11, -0.3)	55.4 (29.9, 78.1)<br>1.1 (-16.4, 18)<br>-1.1 (-16.2, 14.2)	-52.7 (-72.2, -31.2)<br>-0.1 (-16.9, 17-4)<br>-5 (-22.6, 12.2)	12 (11.96, 12.03)<br>75.6 (27.77, 128.67)<br>306.89 (153.79, 423.09)	26.6<br>(0.65, 91.3)
Derryclare	-237.7	3.92<br>(3.72, 4.11)	-1.4<br>(-2.4, 0.4)	-5.5<br>(-13.8, 1.1)	8.8 (-20.3, 37.1)<br>-<br>-	74.6 (54.9, 93.8)<br>-<br>-	12 (11.95, 12.04)<br>-<br>-	29.29<br>(0.93,<br>92.68)

Derrygooney	-267.4	4.09<br>(3.92, 4.27)	-1.9<br>(-3.2, -0.6)	-19.3<br>(-27.2, -11.4)	66 (29.8, 100.3)<br>05.1 (-22.6, 31.6)<br>-	-107.8 (-131.1, -82.8)<br>-11.3 (-36.3, 17.8)<br>-	11.98 (11.95, 12)<br>208.62 (49.52, 379.35)<br>-	18.84<br>(0.14, 88.01)
Dromore	-158.8	3.88<br>(3.7, 4.06)	0.29<br>(-0.64, 1.23)	13.4<br>(-18.6, 7)	8.6 (-25.4, 42.3)<br>-<br>-	-112.7 (-133.9, -90.9)<br>-<br>-	11.97 (11.94,12)<br>-<br>-	23.02<br>(0.63, 89.59)
	-156	3.87<br>(3.67, 4.06)	-0.3<br>(-2.1, 1.2)	-12.6<br>(-19.4, -5.4)	12.7 (-20.7, 45.9)<br>11.6 (-12.5, 350)<br>-	-116.3 (-138.3, -94.6)<br>-23.8 (-57, 13.9)<br>-	11.97 (11.94, 11.99)<br>350.76 (109.99, 433.07)<br>-	22.96<br>(0.5, 90.38)
Egish	-383.3	4.67<br>(4.48, 4.85)	-1.2<br>(-2, -0.4)	-25.4<br>(-33.3, -17.7)	-65.3 (-86.5, -41.3)<br>-<br>-	38.4 (15.5, 59.4)<br>-<br>-	11.97 (11.94, 12.01)<br>-<br>-	23.77<br>(1.11, 90.22)
Emy	9.4	3.67<br>(3.46, 3.86)	2.89<br>(1.22, 4.58)	-25.2<br>(-30, -22.8)	77.6 (34.6, 115.5)<br>-<br>-	-77.3 (-110.2, -39.2)<br>-<br>-	12 (11.92, 12.02)<br>-<br>-	20.87<br>(0.47, 88.36)
	9.8	3.64<br>(3.43, 3.84)	3.1<br>(0.9, 5)	-27.2<br>(-33.6, -20.6)	83.1 (39.8, 120.9)<br>7.4 (-31.8, 41.1)<br>-	-76.2 (-108.9, -37.3)<br>16.5 (-24, 48)<br>-	11.97 (11.93, 12.01)<br>235.84 (65.28, 358.14)<br>-	21.47<br>(0.51, 89.58)
Eske	-537.3	4.41<br>(4.22, 4.61)	-0.1<br>(-1.1, 1)	-6.8<br>(-17.5, 0.9)	23.5 (-2, 47.1)<br>-2 (-18.5, 6.5)<br>-23.6 (-39.7, -3.7)	-57.6 (-73.6, -40.2)<br>-1.6 (-21.1, 17.4)<br>-11 (-31.4, 11.8)	12.01 (11.97, 12.05)<br>77.54 (27.78, 126.37)<br>369.61 (297.79, 413.25)	25.99<br>(0.4, 92)
Fad	-517.9	4.66<br>(4.41, 4.89)	-0.8<br>(-1.6, 0.1)	-16.6<br>(-30.8, -0.9)	29.6 (-2.6, 57.7)<br>-<br>-	-0.049.0 (-68.7, -25.3)<br>-<br>-	12 (11.95, 12.05)<br>-<br>-	25.83<br>(1.4, 91.25)
Feeagh	-510.2	4.28<br>(4.11, 4.49)	-1.6<br>(-2.4, -0.8)	-4.5<br>(-14.1, -0.8)	-49.8 (-73, -24)<br>-<br>-	66.4 (45.4, 85.2)<br>-<br>-	11.98 (11.95, 12.01)<br>-<br>-	27.77<br>(0.48, 92.76)
Fern	22.2	3.31<br>(3.06, 3.54)	-0.3<br>(-2.6, 2.1)	-8.5<br>(-17, -0.02)	-142.2 (-176.5, -105.9)<br>-<br>-	65.7 (10.2, 117.4)<br>-<br>-	12.03 (11.98, 12.08)<br>-<br>-	31.26<br>(1.83, 93.37)
Gartan	-192.4	4.05<br>(3.79, 4.29)	-1.67<br>(-3.37, 0.01)	-11.1<br>(-22.4, -0.2)	-52.1 -80.4, -19.9)<br>-<br>-	-55.2 (-86-4, -17.9)<br>-<br>-	12.03 (11.96, 12.11)<br>-<br>-	20.91<br>(0.21, 89.49)
	-188.6	4.03<br>(3.77, 4.27)	-1.7<br>(-3.7, 0.2)	-11<br>(-22.6, -0.1)	-52.6 (-81.3, -19.2)<br>-1.4 (-29.5, 28.7)<br>-	-58.2 (-89.7, -21.2)<br>5.9 (-29, 38.3)<br>-	12.03 (11.96, 12.1)<br>130.63 (45.83, 272.17)<br>-	22<br>(0.21, 90.31)

Garty	-193.2	4.07<br>(3.82, 4.29)	4<br>(1.4, 6.9)	-31.8<br>(-42.4, -20.9)	19.4 (-31, 67.5)<br>29.6 (-16.5, 66)<br>-	-99.1 (-126.3, -71.5)<br>39.7 (2, 72.6)<br>-	12.02 (11.95, 12.09)<br>234.41 (187.45, 273.84)<br>-	23.97<br>(1.15, 90.13)
Gill	-14	4.1<br>(3.9, 4.32)	-0.3<br>(-1, 0.3)	-10.4<br>(-15.1, -5.6)	-29.7 (-48.1, -9.6)<br>-<br>-	36.8 (20.6, 51.3)<br>-<br>-	11.98 (11.94, 12.02)<br>-<br>-	22.03<br>(25.37, 90.2)
Gleincmurrin	-125.7	3.81<br>(3.62, 4)	-0.1<br>(-1.4, 1.2)	-16.8<br>(-23.6, -10.1)	87.6 (66.6, 108.5)<br>17.8 (-11.8, 40.5)<br>-	3.7 (-27.5, 34.9)<br>9.3 (-20.7, 34.9)<br>-	11.99 (11.95, 12.03)<br>343.52 (74.89, 422.27)<br>-	21.91<br>(0.45, 89.3)
	-125.1	3.83<br>(3.65, 4)	-0.01<br>(-1.08, 1.05)	-18.1<br>(-21.1, -10.4)	85.4 (64.6, 106.1)<br>-<br>-	1.9 (-30, 33.4)<br>-<br>-	11.99 (11.95, 12.03)<br>-<br>-	21.82<br>(0.51, 89.43)
Gowna	39.8	3.81<br>(3.64, 3.99)	-1.8<br>(-2.9, -0.7)	-23.8<br>(-28.8, -18.8)	67.9 (33.5, 100.8)<br>-<br>-	140.7 (108.9, 171)<br>-<br>-	11.97 (11.95, 11.99)<br>-<br>-	24.18<br>(1.12, 90.8)
Inchiquin	-94.4	3.67<br>(3.47, 3.85)	-0.5<br>(-1.8, 0.7)	-9.1<br>(-15.8, -1.8)	-42.9 (-75.7, -8)<br>-8 (-38.9, 37.2)<br>-	-111.8 (-138, -102.8)<br>-13.5 (-3.9, 7)<br>-	11.99 (11.96, 12.02)<br>294.51 (109.14, 418.84)<br>-	21.07<br>(0.2, 89.52)
	-94.2	3.68<br>(3.5, 3.86)	-0.42<br>(-1.46, 0.61)	-9.7<br>(-15.9, -1.3)	-43.9 (-76.5, -9.2)<br>-<br>-	-107.2 (-133.4, -79.5)<br>-<br>-	11.99 (11.96, 12.02)<br>-<br>-	20.14<br>(0.8, 88.74)
Islandeady	-5.9	3.56<br>(3.3, 3.8)	-4.3<br>(-7.4, -1.3)	-16.5<br>(-26.1,-5.9)	-18.7 (-70.6, 34.6)<br>-<br>-	-133.7 (-172.7, -95)<br>-<br>-	12.03 (11.96, 12.1)<br>-<br>-	22.48<br>(0.38, 90.38)
	-1.9	3.49<br>(3.23, 3.75)	-4.6<br>(-8.33, -0.71)	-17.3<br>(-24.5,-6.5)	-10.8 (-63.8, 43.9)<br>15.6 (-34.6, 58.8)<br>-13.1 (-46.2, 22)	-149.1 (-189.1, -108.4)<br>28.8 (-25.8, 69.7)<br>-10.1 (-45.5, 31.2)	12.03 (11.96, 12.09)<br>74.05 (44.61, 112.28)<br>194.57 (146.95, 234.25)	23.79<br>(0.36, 90.34)
Lickeen	-141.4	4.33<br>(4.1, 4.54)	-3.4<br>(-5.3, -1.7)	-20.8<br>(-28.6, -13.1)	-62.4 (-81.5, -42.8)<br>35.1 (8.1, 57.3)<br>-	0.7 (-28.8, 30)<br>8.9 (-32.7, 44.9)<br>-	12.01 (11.95, 12.07)<br>272.42 (219.52, 327.87)<br>-	22.54<br>(0.8, 89.68)
Muckno	93.1	3.77<br>(3.6, 3.94)	-2.6<br>(-3.74, -1.47)	-19.6<br>(-22.2, -14.9)	-74.9 (-95, -53.4)<br>-<br>-	10.6 (-20.3, 40.9)<br>-<br>-	11.99 (11.94, 12.04)<br>-<br>-	18.94<br>(0.16, 87.52)
	95.8	3.76	-2.6	-19	-75.8 (-96, -54.8)	10 (-21.4, 40.7)	11.99 (11.94, 12.04)	19.05

		(3.58, 3.93)	(-3.8, -1.1)	(-23.9, -14)	-6.8 (-29.3, 21.7)<br>-	-0.6 (-25.5, 28.6)<br>-	253.45 (43.44, 426.77)<br>-	(0.16, 87.76)
Nadregeel	-160.5	4.17<br>(3.95, 4.38)	-1.1<br>(-2.5, 0.5)	-0.2<br>(-28.3, -11.7)	-23.5 (-56.1, 10.2)<br>-7.2 (-28.8, 16.8)<br>-	-108.2 (-134.3, -89)<br>-0.8 (-24.2, 26.3)<br>-	12.01 (11.97, 12.05)<br>218.52 (33.95, 345.55)<br>-	20.1<br>(0.28, 29.13)
	-158.4	4.19<br>(3.97, 4.42)	-1.16<br>(-2.38, 0.07)	-0.28<br>(-27.8, -12.6)	-24.763 (-57.1, 8.9)<br>-<br>-	-106.031 (-132.4, -78.8)<br>-<br>-	12.01 (11.97, 12.04)<br>-<br>-	20.09<br>(0.31, 89.37)
Oughter	527.3	2.74<br>(2.56, 2.9)	-1.5<br>(-3.1, 0.1)	-14.5<br>(-18.7, -10.5)	116.2 (58.4, 170.7)<br>-<br>-	-207 (-242.3, -170.1)<br>-<br>-	11.98 (11.96, 12)<br>-<br>-	20.86<br>(0.23, 88.13)
Sillan	-192.3	4.06<br>(3.87, 4.25)	-0.3<br>(-1.4, 0.8)	-21.1<br>(-27.9, -14.3)	-13.2 (-47.9, 21.5)<br>-<br>-	-126.7 (-150.8, -102.3)<br>-<br>-	12 (11.97, 12.03)<br>-<br>-	19.73<br>(0.23, 87.89)
Skeagh	-238	4.72<br>(4.51, 4.91)	-0.24<br>(-0.82, 0.35)	-21.8<br>(-25.5, -13.1)	32.3 (15.3, 46.4)<br>-<br>-	30.1 (8.6, 49)<br>-<br>-	11.99 (11.95, 12.03)<br>-<br>-	23.76<br>(1.19, 90.41)
	-234.4	4.71<br>(4.48, 4.91)	-0.3<br>(-1, 0.4)	-20.9<br>(-27.6, -14.3)	32.2 (15.2, 46.6)<br>3.5 (-8.8, 15.4)<br>-	30.7 (9.2, 49.5)<br>0.6 (-13.8, 13.5)<br>-	11.99 (11.95, 12.03)<br>254.66 (39.11, 435.28)<br>-	23.82<br>(1.71, 89.95)
White	54.7	3.45<br>(3.26, 3.63)	-0.29<br>(-1.59, 0.99)	-16.3<br>(-20.8, -8.2)	-64.6 (-106.1, -20.7)<br>-<br>-	130.4 (102, 157.5)<br>-<br>-	11.97 (11.94, 12)<br>-<br>-	23.76<br>(0.69, 89.84)
	54.7	3.45<br>(3.26, 3.63)	-1<br>(-3.2, 0.9)	-15.1<br>(-21, -9.1)	-66.8 (-107.1, -22.9)<br>10 (-19.8, 37.3)<br>-	131.9 (103.2, 158.8)<br>-16.2 (-60.4, 30.5)<br>-	11.97 (11.94, 12)<br>278.2 (53.83, 402.14)<br>-	23.5<br>(0.58, 90.63)
